# Granite classification using machine learning and edge computing

**DOI:** 10.12688/f1000research.124057.1

**Published:** 2022-11-08

**Authors:** Madhavi Karanam, Krishna Chythanya Nagaraju, Gotham Sai P, SaiKiran Manasa S, Pranay Krishna G

**Affiliations:** 1 Professor,HoD,CSE,, Gokaraju Rangaraju Institute of Engineering and Technology, Hyderabad, Telangana, 500090, India; 2 Asst.Prof., CSE, Gokaraju Rangarau Institute of Engineering and Technology, Hyderabad, Telangana, 500090, India; 3 UG STUDENT, CSE., Gokaraju Rangaraju Institute of Engineering and Technology, Hyderabad, Telangana, 500090, India

**Keywords:** Granite, Machine Leaning, Edge Computing, Random Forest, SVM, TCS3200, ESP8266

## Abstract

Background: The outlook and the aura of any place are highly dependent on how a place is decorated and what materials are used in designing it. Granite is such a kind of rock which is vastly used for this purpose. Granite flooring and countershave a major influence on the interior d ´ecor which is essential to set the moodand ambience of a house. A system is needed to help the end users differentiatebetween granites, which enhance the grandeur of their house and also check thefrauds of different color granite being sent by the merchant as compared to whatwas selected by the end user. Several models have been developed for this causeusing CNN and other image processing techniques. However, a solution for thispurpose must be precise and computationally efficient. Methods: For this purpose,researchers in this work developed a machine learning based granite classifier us-ing Edge Computing and a website to help users in choosing which granite wouldgo well with their d ´ecor is also built. The developed system consists of a colorsensor [TCS3200] integrated with an ESP8266 board. The data pertaining to RGBcontrasts of different rocks is acquired by using the color sensor from a dealership.This data is used to train a Machine Learning algorithm to classify the rock intodifferent granite types from a granite dealer and yield the category prediction. Re-sults: The proposed system yields a result of 94% accuracy when classified usingRandom Forest Algorithm. Conclusion: Thus, this system provides an upper handfor the end users in differentiating between different types of granites.

## Introduction

Granite is a coarse-grained igneous rock. It is formed when magma is compressed due to the pressure underground. It is one of the most common rocks found on earth crust and constitutes a multimillion dollar granite industry. Granite owing to its beauty and composition, it is used in a wide range of applications which encompass house decor, flooring, counter tops etc. This flexibility of granite has instigated many people to cheat with the quality and type of granite. With the advancing technologies, like machine learning and Internet of Things (IOT) the verification and analysis of such samples has become an easy task. These technologies have made it possible to go about any particular task in an efficient and remote manner with pinpoint precision. This has led some researchers to explore the field and produce some workable solutions, models which recognize the type of granite using Convolutional Neural Network (CNN) on images of granite. These models classify the granites based on image patterns of different types of granite. ^1^

Models which use Computer Vision to classify rocks using the colors and textures of granites obtained from images. This model considers the color of granite and it’s texture as the distinguishing factor. ^2^ Models were developed which apply Machine Learning on data gathered using a spectrophotometer. These models gather data from a spectrophotometer and then apply Machine Learning to differentiate between granite types. ^3^

The solutions currently developed all rely on heavy computing techniques and are not portable. A solution which can be executed from anywhere at any time needs to be developed to better cater the needs of end users. To meet the demands of end users and make the system more portable and efficient, a model with Machine Learning and Edge Computing is experimented in this work. The increasing standards of living and per capita income have also inspired people to try different styles and experience various luxuries, primarily the looks of their houses. This sudden inflow of income and need for granite has instigated some merchants to fraud the end users by charging for a higher quality rock and issuing a lower quality rock. With these frauds on the incline, the financial situations are on a decline. It’s high time that we develop a method to verify the quality of the rock and terminate the frauds. The end users also often seek suggestions to choose from the plethora of granites that are available in the market. It is important now more than ever to devise a solution to corroborate the granite received in order to prevent the fraud efficiently and effectively without compromise on the portability. The integration of ML and Edge Computing is an effective way to do the required task much more easily.

This project utilizes the color variations of granites to classify them into various categories by collecting the color values from separate points on the rock and applying Machine Learning on them. The major objective of this work is to verify the rock delivered based on color.

## Previous work

The homogeneity of pieces of granites being used based on color is very important for the aesthetic of place where granites are being used. In general humans carry out the classification process based on quality and look using human experience and exposure along with a bit of creativity. This opens doors for artificial intelligence based works to be carried out in this field in selection of a specific color granite.

Reference 1 work follows convolution neural network usage for granite classification. The researchers used transfer learning on MNSIT networks and CIFAR networks on a dataset of 1000 RGB images. Using Nearest Neighbor classifier with CIFAR they claim to achieve an accuracy of above 85% in classifying granites.

In the work ^2^ an expert system was developed based on computer vision concepts to classify granites. The authors claim that Support Vector Machine out performs than other methods they tried in classifying granites based on color and texture. The classification of granites using automatic artificial intelligence based systems would provide benefits of creating a repeatable procedure which is not possible in manual general process followed for decades in market. This further add to substantial upgrading in quality assessment procedures for companies. Also, lessening of losses owing to cancellation of order at customer site besides easy warehouse management can be achieved. The work was carried out using lot of 48 granites of twelve classes on total which were subdivided in to 64 samples per class and images were captured using a standard procedure in lab but finally the cropped image of “544×544 pixels” was used in experimentation. They used methods based on co occurance matrix, Gobor features, chromatic features and Local Binary Patterns. The work made use of multiple classifiers including “Support Vector classifier”, “Linear Classifier” “Naïve Bayes’”, “Nearest Neighbor” etc.

In some works sum and differences of histograms along with LVQ neural networks is used to classify granites. ^4^ In the letter published by Ref. 5 the researchers claim to use “wavelet analysis” to classify the granites. Discrete wavelet transformation is used to generate sequences of signals in their work. The researchers made use of 30 image database having three classes of granites. The work claims to obtain 90% accuracy using LVQ neural networks.

With the work ^6^ the researchers have proposed an “electro-mechanical system” that robotically categorizes marbles while on conveyor belt itself based on a “hierarchical clustering approach”. They made use of Programmable Logic Controller along with an auxiliary micro controller to bind between PLC and Matlab. Clustering based classification was implemented along with hierarchical classification based on cascaded features of color, texture and spectral. 83.6% accuracy as recorded in their work for correct classification of marbles. Reference 7 work makes use of transfer learning on AlexNet and VGGNet to develop a Convolution Neural Network based granite tiles classification using a dataset of 2000 RGB images classified into 25 classes. Out of experiments they observed that fine tuning VGGNet they could record an accuracy of almost 99.3%.

The authors of Ref. 8 have implemented there own CNN architecture and good bring out an accuracy of around 96.1% in marble quality classification. The authors of Ref. 9 have done extensive experimentation by examining fifteen varieties of convolution neural networks to sort dolomitic stone tiles. They observed that “DenseNet201” model could yield an accuracy of 83.24% while trained on a 489 digital image data set of granites classified into 3 classes.

## Methods

The Model developed takes the color values of granite as input and then feeds it to a machine learning algorithm. The sensor relays data to a nodemcu, which analyses it and deploys a machine learning model to verify the granite being sampled and classifies it into a specific class of rock.

### Machine learning

**Figure 1.**
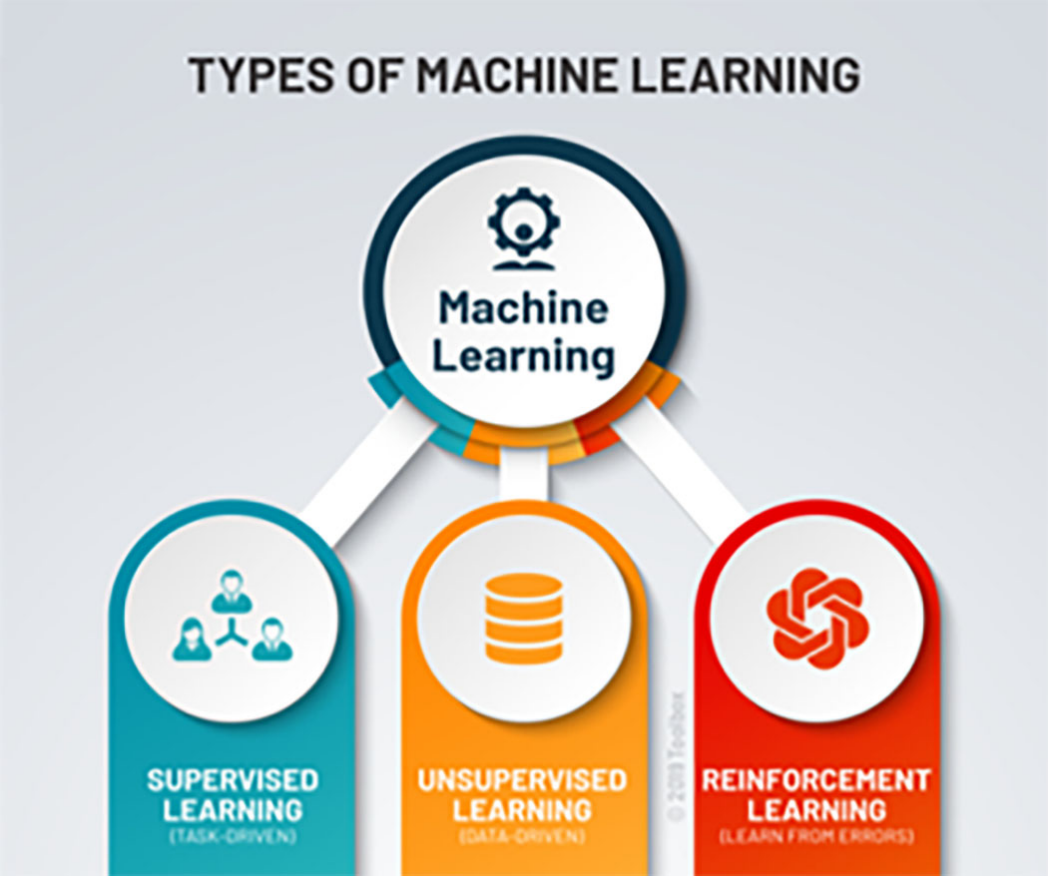
Types of machine learning.

Machine learning is a part of Artificial Intelligence (AI) that trains a computing machine to learn from data and improve. The term machine learning depicts an automated process of pattern recognition and feature detection in any data. Machine learning also increased the comforts of living, personal assistants are now available which cater to a person’s personal needs and desires. Machine learning can be categorised as Supervised machine learning, Unsupervised machine learning, Semisupervised learning as shown in Figure 1 below.

**Figure 2.**
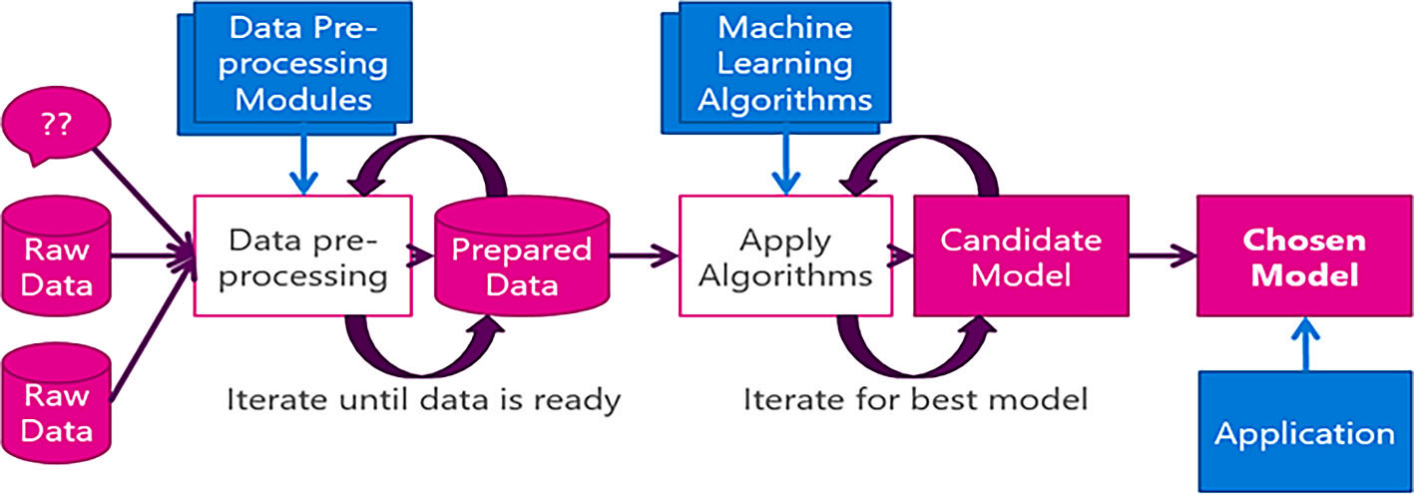
Machine learning process.

Machine learning models require a large amount of data to be trained on for efficient prediction and increased accuracy. The data can be obtained from a variety of methods including real time data collection as done in this work. This data can be processed in a lot of ways such as normalization, removing data or filling the missing data with mean, median or mode and other ways. After processing the data, the Machine learning models are ready to be trained. The authors opted for classification model of ML to be implemented for this work as objective is to classify granites based on color. There are several Algorithms which analyze the data and predict/classify the output. Each of them works on a different principle and the ML model. Training is the part where the machine learns the data and analyses it. The Output and the efficiency majorly depend on this part. The data is trained to provide a specific output depending on the input provided. Evaluation of a model refers to the part where the model’s efficiency is tested. In this work Accuracy and Confusion matrix are considered to evaluate trained model. The overall machine learning process discussed can be depicted in figure as shown in Figure 2.

Edge computing refers to a distributed computing paradigm, in which the data is stored and computations are executed closer to the actual site in order to improve response time and carry the functions of any process faster. It’s more of a topology. The purpose of Edge computing is to move the entire computation of any process away from data centers and closer to the edge of the network in order to reduce the stress and load on the data center for efficient and quicker responses. It exploits the functions and specifications of smart objects like smart phones, controller boards etc., which have built-in memory to perform tasks and provide services instantaneously rather than accessing the data centers and retrieving information for every task.

### Dataset

**Figure 3.**
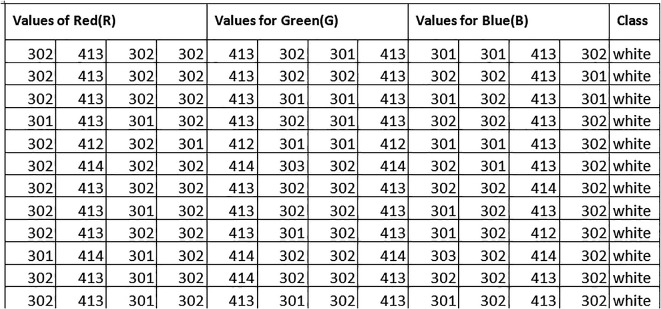
The sample of data set used for in this experiment.

Dataset used is collected from a granite dealership with the consent of pertaining dealers in real time. This collected data is then used to train the developed IoT based machine learning model using edge computing. The Features of the dataset include RGB values of different types of granites and the last column giving the color of granite. The sample of data set collected is shown in Figure 3 below.

The sensors were taken to a dealership and the RGB values pertaining to the different classes of granites are recorded. This data is then stored in a csv file for easier training of ML models.

### Data collection

**Figure 4.**
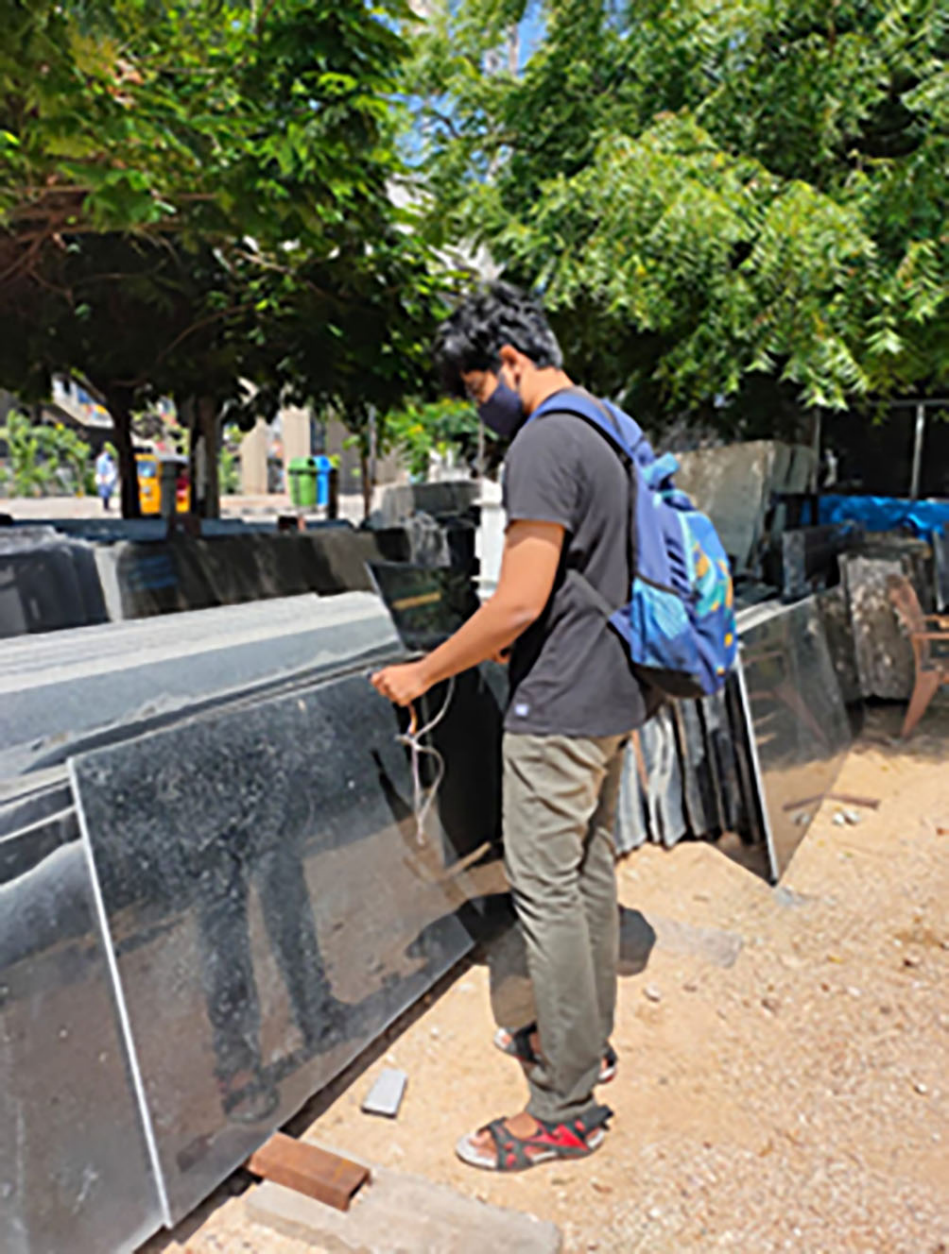
One of the authors collecting data using sensors.

**Figure 5.**
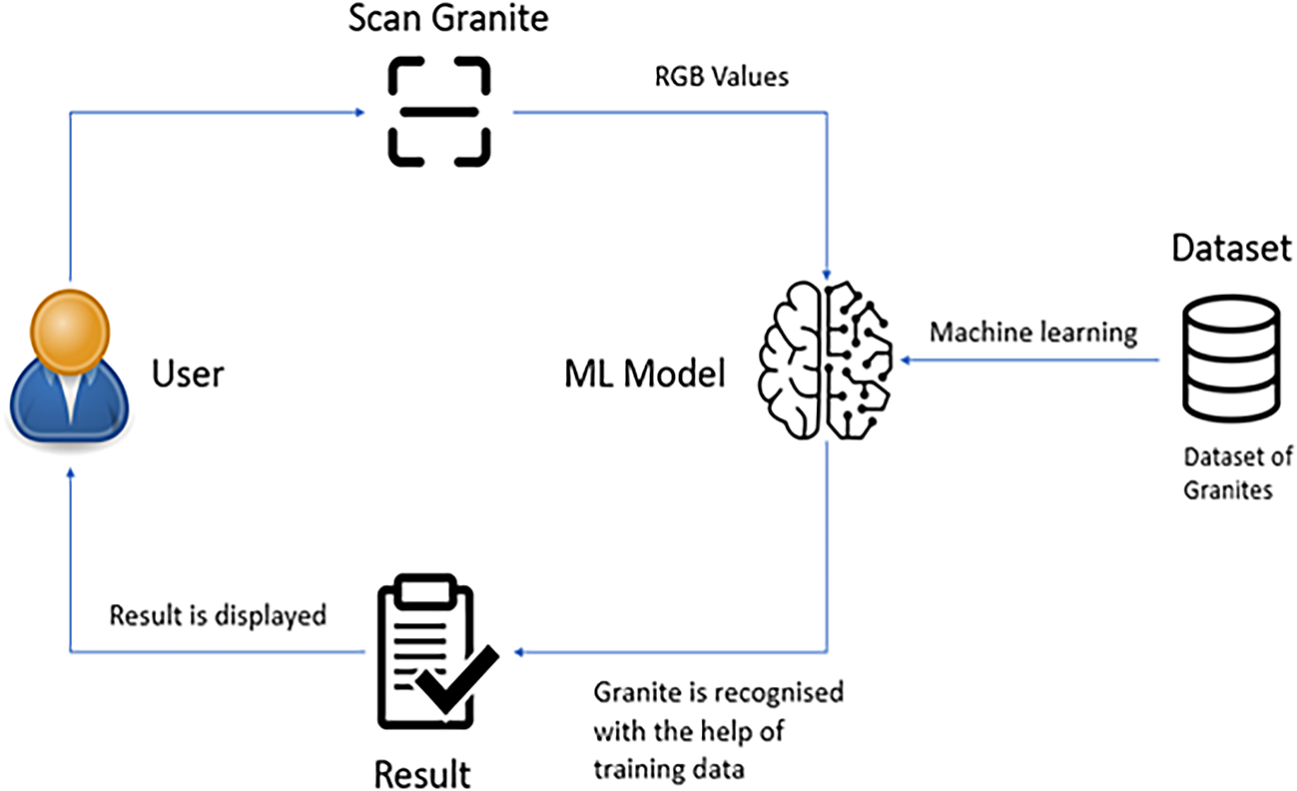
Architecture of the system implemented.

The ESP8266 and color sensor were integrated to build the experimental setup for the purpose of recording the RGB values of granites and for the collection of dataset. A dealer was contacted to get the permission for recording the values of granites and after taking necessary precautions and permission. The values of red granite, black granite and white granite were collected and tabulated as shown in Figure 3 below. The data was collected by one of the authors of this work as can be found in image of Figure 4 below. The dataset acquired accommodates 90 rows with 12 columns of rgb values describing 3 classes. A program was written in Arduino IDE to collect the sensor readings. The sensor gives “rgb” values at each point by adjusting the s0 and s1 pins. These RGB values are stored in an array. The seuence diagram of idea carried out can be seen in the figure below. The overall system architecture of the idea implemented can be seen as in Figure 5 below. This work is implemented using Arduino IDE for edge computing, python language to perform machine learning and HTML, CSS to develop the web application. With the help of special libraries, machine learning is carried out on the Arduino platform. The Arduino (IDE) software makes the uploading of code to the NodeMCU much easier and quicker. Python Language is used to implement the machine learning modules using Scikit Learn library which is then transferred to Arduino.

The Dataset built already is given to train machine learning models to learn. After the data is trained and models learnt the process, various algorithms are evaluated and compared to select the one with highest accuracy for classification i.e. Random forest. The Implementation of this project is done in 3 steps: 1. Data collection: collection of data from the granite dealership; 2. Model Selection: various ML algorithms were observed and a model is fixed; 3. Deployment: The model is deployed into a nodemcu for real time usage.

Various machine learning algorithms are used to classify any particular data and each one of them is dependent on a specific mathematical concept. These concepts are then applied to find a most suitable correlation with the given data or a function that best fits the data being analyzed. The algorithms that are compared for better output in this work are: Random Forests Algorithm ^10^; Support Vector Machine Algorithm ^11^; K-Nearest Neighbors (KNN) Algorithm. ^12^ The algorithms are implemented in python and then converted to C for it to be executed in NodeMCU. NodeMCU is an open-source firmware, used for IoT applications on open source boards. Lua scripting language is used to implement the firmware on Espressif SDK for Esp8266. Esp8266 image is shown in figure below. A Wi-fi soc from espressif systems is available on the board. A dual in-line package provided by the prototyping hardware integrates a USB controller with an MCU and antenna laden board. UART, DAC and ADC interfaces are supported by the board and can be accessed through a specific set of pins among the 21 pins available on the board.

The deployment is done using python IDLE and Arduino IDE. Micromlgen is an open source library developed to bring machine learning to microcontrollers. It essentially converts a ML code into an optimized c code for the microcontrollers to execute it. This acts as an alternative to the Tensorflow package which is computationally complex and cannot be used on boards with smaller capacities and memories. Micromlgen supports some of the basic machine learning algorithms like SVM, Random Forests, Decision trees, Gaussian Naive Bayes, XGBoost among others. The obtained output code in C is then written in a header file which is to be included in the arduino sketch. Thus, ML is implemented in the NodeMCU effectively. The sensor is then connected to the NodeMCU and the model is loaded onto the board. The physical model gets ready and can be used to classify granites in real time.

**Figure 6.**
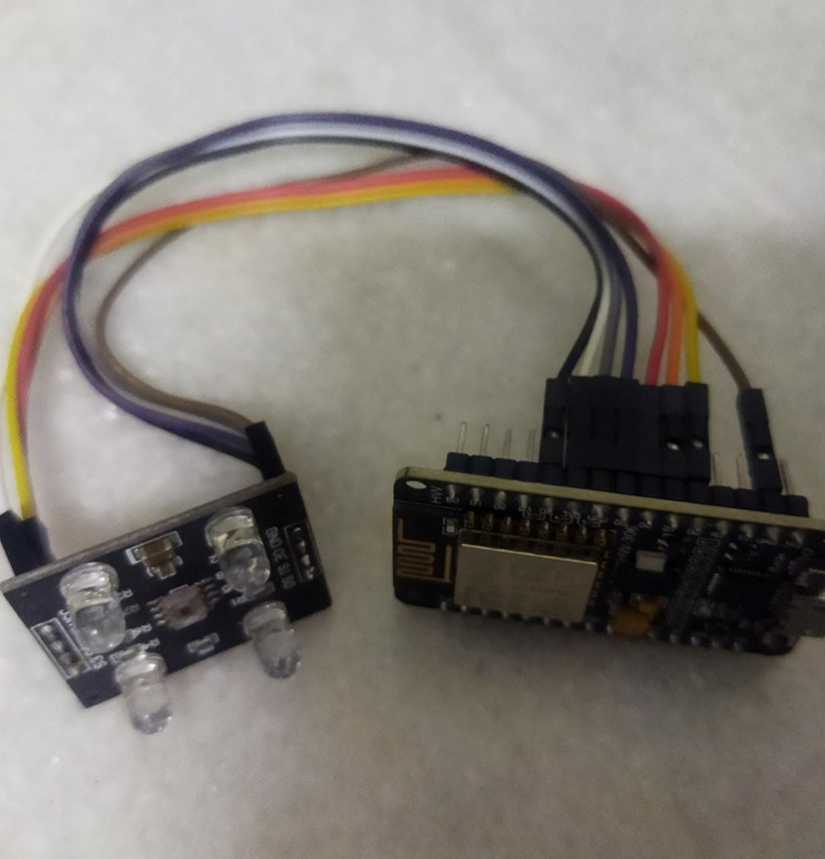
SetUp for Edge Computing using ESP8266 board and TCS3200 used for implementing this work.

TCS3200 color sensor contains a TAOS TCS3200 RGB sensor chip and 4 white LEDs. The image of TCS3200 color sensor is shown in the image in figure given below. The chip forms the most important module of the sensor, which is basically a color light-to-frequency converter. The sensor is arduino compatible, which makes it very useful and easy to handle. The corresponding frequency of various colors is given as output by sensing the presence of Red, Green and Blue colors. This module can be used in a variety of applications such as granite classification, color matching tests, color sorting robots, etc. The setup built for Edge Computing using ESP8266 and TCS3200 for carrying out the idea can be seen in the image of Figure 6 below.

**Figure 7.**
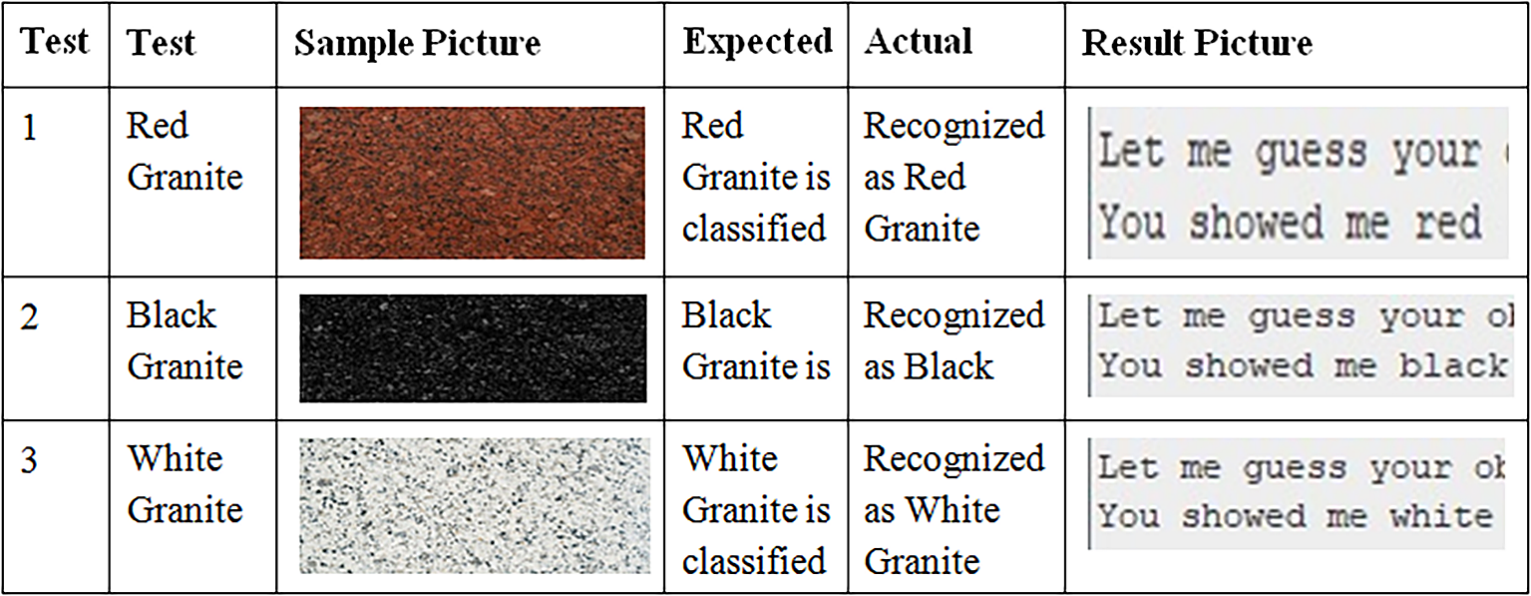
Sample outputs for the test inputs given.

The Figure 7 above shows the sample results found during experimentation.

**Table 1.** Different algorithms tried and their accuracy.

Algorithm	Accuracy
Random forest	94%
SVM	78%
KNN	75%

## Conclusion

In this work the model built helps the users in choosing a granite that enhances the grandeur of their house and also reduces losses for return orders at merchant end. The model aims to help the end users in ascertaining the quality and type of granite they received. Improvising the age- old techniques of image classification, this model does the task with edge computing for increased efficiency and effectiveness. The researchers tested the dataset using several machine learning algorithms which have produced satisfactory results. Random Forest classifier gave 94% accuracy, SVM exhibited 78% accuracy and KNN could only achieve 75% accuracy. The values have been shown tabular form in Table 1 above. Random Forest algorithm yielded the best results. The accuracy can be improved by collecting larger data.

## Author contributions

Mrs. K. Madhavi was chief mentor for the work and supervised the whole research taken up with time to time inputs. She took care of reviewing the article and methodology.

Mr. Krishna Chythanya conceptualised the idea and assisted in Implementing the research with proper evaluations time to time and also writing the article.

Mr. P. Gowtham Sai was instrumental in collecting data as well as implementing the code.

Mr. S. Saikiran Manasa was contributing in algorithm implementation and evaluation.

Mr. G. Pranay Krishna was playing role during documentation and review.

## Data availability

Figshare. dataset *_G_ raniteClassi f ication.* *csv.* DOI: 10.6084/m9.figshare.20301006.

This project contains the following underlying data:

‐ The data set has around 90 rows each consisting of 12 coloumns that stands of RGB values of Granite. Each of RGB is represented by 4 values respectively in a row. These values are recorded using TCS3200 color sensor on granite peices. The values were recorded by visiting couple of near by granite dealer shops in Hyderabad, Telangana, India. The last column denotes the color of granite and thisacts as a target variable in case of machine learning algorithms to predict the color of granite.

## Source code

Zenodo. GRANITE CLASSIFICATION USING MACHINE LEARNING AND EDGE COMPUTING. DOI: 10.5281/zenodo.6835324.

‐ The RAR file consists of three folders. Each folder has got code for each of the three machine learning algorithms implemented for Granite Classification using Edge Computing. The code has folders by name KNN, SVM and Random Forest.

## References

[1] Ferreira A Giraldi G : Convolutional neural network approaches to granite tiles classification. *Expert Syst. Appl.* 2017;84:1–11. 10.1016/j.eswa.2017.04.053

[2] Bianconi F González E Fernández A : Automatic classification of granite tiles through colour and texture features. *Expert Syst. Appl.* 2012;39(12):11212–11218. 10.1016/j.eswa.2012.03.052

[3] Araújo M Martínez J Ordóñez C : Identification of granite varieties from colour spectrum data. *Sensors.* 2010;10(9):8572–8584. 10.3390/s100908572 22163673 PMC3231240

[4] Tomás-Balibrea L-M : Automatic classification system of marble slabs in production line according to texture and color using artificial neural networks. *International Conference on Computer Analysis of Images and Patterns.* Springer;1999; pp.167–174.

[5] Luis-Delgado JD Martinez-Alajarin J Tomas-Balibrea LM : Classification of marble surfaces using wavelets. *Electron. Lett.* 2003;39(9):714. 10.1049/el:20030496

[6] Alper Selver M Akay O Alim F : An automated industrial conveyor belt system using image processing and hierarchical clustering for classifying marble slabs. *Robot. Comput. Integr. Manuf.* 2011;27(1):164–176. 10.1016/j.rcim.2010.07.004

[7] Ather M Khan B Wang Z : Automatic recognition and classification of granite tiles using convolutional neural networks (cnn). *Proceedings of the 2019 3rd International Conference on Advances in Artificial Intelligence.* 2019; pages193–197.

[8] Karaali I Eminağaoğlu M : A convolutional neural network model for marble quality classification. *SN Appl. Sci.* 2020;2(10):1–6. 10.1007/s42452-020-03520-5

[9] Ouzounis AG Sidiropoulos GK Papakostas GA : Interpretable deep learning for marble tiles sorting. *DeLTA.* 2021;101–108.

[10] Ho TK : Random decision forests. *Proceedings of 3rd international conference on document analysis and recognition.* Vol.1. IEEE;1995; pp.278–282.

[11] Meyer D Wien FT : Support vector machines. *R News.* 2001;1(3):23–26.

[12] Peterson LE : K-nearest neighbor. *Scholarpedia.* 2009;4(2):1883. 10.4249/scholarpedia.1883

