# Supplementary material for: Granite classification using machine learning and edge computing
Source: F1000Res. 2022 Nov 8;11:1276. [Version 1] doi: 10.12688/f1000research.124057.1 (PMC10683781; doi:10.12688/f1000research.124057.1)
Supplement: Peer review history

# Reviewer report: Reviewer response for version 1

**DOI:** 10.5256/f1000research.136228.r225149

**Published:** 2023-11-27

**Authors:** Zhi Yu

**Affiliations:** 1 Zijin School of Geology and Mining, Fuzhou University, Fuzhou, China

1. The abstract requires revision, with the removal of specific terms such as background, methods, and results.

 2. Clarify the term "d’ecor?" in the abstract.

 3. Provide an introduction to the development of the machine learning model.

 4. Introduce the data preprocessing steps and specify the ratio of training to testing datasets.

 5. Include a comparative analysis of model performance.

 6. Add sections on limitations and future scope.

 7. In the introduction, highlight the limitations of prior studies and underscore the significance of this paper.

 8. Enhance the quality of the figures presented in the paper.

 9. Reorganize the content of the article for better flow.

 10. Clarify the content of Figure 7, specifically the phrase "Lest me guess you?"

Is the work clearly and accurately presented and does it cite the current literature?

Yes

If applicable, is the statistical analysis and its interpretation appropriate?

Partly

Are all the source data underlying the results available to ensure full reproducibility?

Partly

Is the study design appropriate and is the work technically sound?

Yes

Are the conclusions drawn adequately supported by the results?

Yes

Are sufficient details of methods and analysis provided to allow replication by others?

No

Reviewer Expertise:

Mining Engineering; Application of machine learning technique

I confirm that I have read this submission and believe that I have an appropriate level of expertise to confirm that it is of an acceptable scientific standard, however I have significant reservations, as outlined above.

# Reviewer report: Reviewer response for version 1

**DOI:** 10.5256/f1000research.136228.r225152

**Published:** 2023-11-27

**Authors:** Blessing Olamide Taiwo

**Affiliations:** 1 Federal University of Technology Akure, Akure, Ondo, Nigeria

Firstly, I want to thank the authors for putting this great work together and for their efforts.

 The manuscript presents the development of machine learning based granite classifier using Edge Computing and a website to help users in choosing which granite would go well with their decor is also built. The authors considered color sensor [TCS3200] integrated with an ESP8266 board in the work.

 I have the following comments and recommendation to improve the current state of the work.

 1. Grammar and Presentation:

 The work needs some rephrasing and corrections to improve the grammar and presentation. (Pg 4 last paragraph and other areas).

 2. Literature Review:

 The author provides some literature about previous work, but i recommend they include recent works relating to optimization algorithms, ensemble learning classifiers to provide more ground to users about the methodology used in this work.

 3. Model Result validation and Data description:

 I recommend that the author should provide the data in Table form and also provide the testing input dataset along with Figure 7.

 The use of ROC as part of the result assessment should be included in the result section.

 " **ROC curve** is a graphical representation of the performance of a binary classifier system as its discrimination threshold is varied. It is created by plotting the **true positive rate (TPR)** against the **false positive rate (FPR)** at various threshold settings."

 Authors should provide the chart for both FPR and TPR for both training data only for testing data to enhance the result interpretation.

Is the work clearly and accurately presented and does it cite the current literature?

Partly

If applicable, is the statistical analysis and its interpretation appropriate?

Partly

Are all the source data underlying the results available to ensure full reproducibility?

Partly

Is the study design appropriate and is the work technically sound?

Yes

Are the conclusions drawn adequately supported by the results?

Yes

Are sufficient details of methods and analysis provided to allow replication by others?

Partly

Reviewer Expertise:

Engineering application of Artificial intelligence

I confirm that I have read this submission and believe that I have an appropriate level of expertise to confirm that it is of an acceptable scientific standard, however I have significant reservations, as outlined above.

# Reviewer report: Reviewer response for version 1

**DOI:** 10.5256/f1000research.136228.r157142

**Published:** 2022-12-16

**Authors:** Ratan Lal

**Affiliations:** 1 Northwest Missouri State University, Maryville, MO, USA

The authors consider the problem of identifying the quality of Granite rock to help people and avoid fraud in Granite rocks. The broad approach is the following. First, a set of RGB colors for real images is collected through the color sensors. Then, the data has been used to train different machine learning models, such as Random Forest, Support Vector Machine, and K Nearest Neighbor. Upon training the model, the best among the selected models has been considered to deploy on NodeMCU, which is an open-source firmware used for IoT applications on open-source boards. The model is trained through Scikit Learn Library in python, and then it is converted into C using Micromlgen in order to deploy the ML model on the NodeMCU. Finally, the Random Forest model has 94% accuracy, and it is deployed on the NodeMCU.

 Pros:

1. The idea of identifying the quality of Granite rock using IoT devices is interesting.
2. The paper is written well.

Major Comments:

1. I recommend the authors use the term “prediction” in place of “verification” as the machine learning model does not always give an accurate result.
2. It would be good if the authors could report the confusion matrix for the Random Forest model.
3. I recommend the authors remove methods:, results: heading and conclusion: paragraph from the abstract.

Minor comments:

1. On page 6, seuence -> sequence
2. On page 7, python IDLE -> python IDE

Is the work clearly and accurately presented and does it cite the current literature?

Yes

If applicable, is the statistical analysis and its interpretation appropriate?

Yes

Are all the source data underlying the results available to ensure full reproducibility?

No

Is the study design appropriate and is the work technically sound?

Yes

Are the conclusions drawn adequately supported by the results?

Yes

Are sufficient details of methods and analysis provided to allow replication by others?

Yes

Reviewer Expertise:

Formal Verification, Cyber-Physical Systems, Robotics, Machine Learning, Internet of Things

I confirm that I have read this submission and believe that I have an appropriate level of expertise to confirm that it is of an acceptable scientific standard.
